# Protocol for integrated analysis of bacterial RNA-seq and ChIP-seq data for establishing a co-expression network

**DOI:** 10.1016/j.xpro.2023.102322

**Published:** 2023-05-19

**Authors:** Yiqing Ding, Jingwei Li, Tianmin Li, Xin Deng

**Affiliations:** 1 Department of Biomedical Science, City University of Hong Kong, Kowloon Tong, Hong Kong SAR, China; 2 Shenzhen Research Institute, City University of Hong Kong, Shenzhen 518057, China

**Keywords:** Genetics, Microbiology

## Abstract

Transcriptomic data and ChIP-seq data from bacteria are systematically analyzed and efficiently combined. We describe the software environment for analysis and the download and installation methods. Furthermore, we describe the analytical process and present the corresponding mini-test data, which can be conveniently restored and reproduced by users. Moreover, we provide the script for data consolidation, which allows multiple files to be rapidly merged. Overall, this protocol presents software parameters, R codes, and in-house Perl scripts for analyzing bacterial multi-omics data.

For complete details on the use and execution of this protocol, please refer to Xin et al.

## Highlights

• Protocol for integrated analysis of bacterial RNA-seq and ChIP-seq data
• R language code for easy data visualization and figure graphing
• In-house scripts help to process intermediate text, making the analysis streamlined
• Mini data for sequencing and genomes for preliminary simulated analyses

## Abstract (editor highlights)

Publisher’s note: Undertaking any experimental protocol requires adherence to local institutional guidelines for laboratory safety and ethics.

## Graphical abstract

**Figure:**
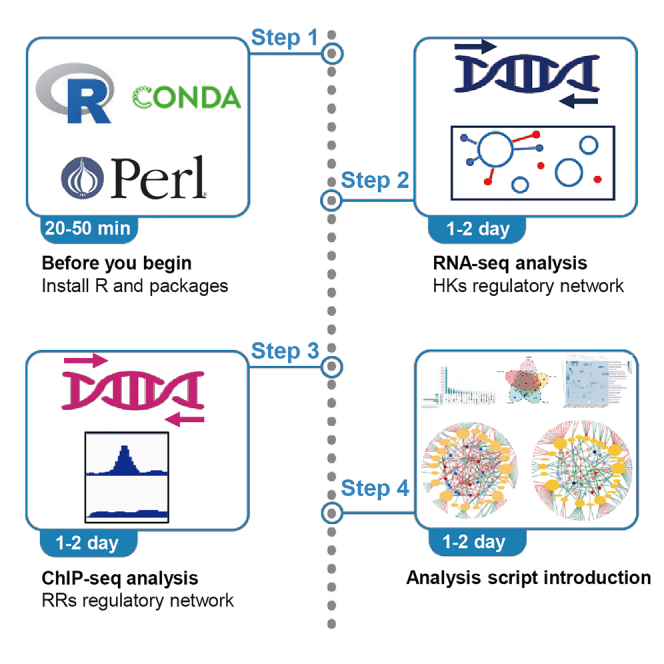

## Before you begin

Before you begin your analysis, you need to prepare a platform for data storage and analysis. This procedure is performed on Linux CentOS, and you can download software and R packages online. The memory of the platform should be at least 10 times the size of the genome data.

Processing of the ChIP-seq data is initiated with a quality check to ensure that the data do not contain the artificial sequences typically added during the experiment. The data filtered by removing adapter and primer sequences and low-quality reads are typically referred to as clean data. In the subsequent analysis, we use only clean data. After using Bowtie2^1^ and Samtools^2^ to translate the clean data into the genome, the Model-Based Analysis of ChIP-Seq (MACS)^3^ framework is used to identify peaks. Bowtie2 is an ultrafast in-province tool for matching short sequences to template genomes. Bowtie2 uses the Burrows–Wheeler transform to establish the index for the genome, which has a small memory footprint and is convenient for rapid comparison. MACS is one of the most widely used peak-calling programs, which improves the spatial resolution by combining information from two sequencing tags to integrate the site location and orientation. The ChIPpeakAnno^4^ package is used to perform the downstream analysis after peak calling, including searching for the genes closest to the peak region, gene ontology (GO) enrichment analysis of the peak adjacent genes, and extraction of the sequences of the peak and its surrounding regions. Spliced Transcripts Alignment to a Reference (STAR) is used to map the transcriptome data to the genome, HTSeq^5^ is used to obtain the raw counts, and DESeq2^6^ is used to determine the differential genes. In conclusion, the protocol uses the mainstream software and scripts to improve the ease and efficiency of ChIP-seq and RNA-seq data processing.

### Install required software

**Timing: 10–30 min**

Install Miniconda and the software required by ChIP-seq and RNA-seq.

1. Install Miniconda.

Miniconda^7^ is a free minimal installer for Conda that includes only Conda, Python, the packages they depend on, and a small number of other useful packages.

***Note:*** Bioconda is a channel on Conda that distributes bioinformation software and currently includes more than 2,700 apps. Bioconda can be used to search for available software on this channel.

> wget -chttps://mirrors.bfsu.edu.cn/anaconda/miniconda/Miniconda3-latest-Linux-x86_64.sh

> bash Miniconda3-latest-Linux-x86_64.sh

> conda config --add channels conda-forge

> conda config --add channels r

> conda config --add channels bioconda

> conda config --add channels defaults

2. Install the software required by ChIP-seq.

FastQC, Bowtie2, Samtools, and MACS are required for ChIP-seq analysis.

***Note:*** FastQC^8^ is a quality control tool for high-throughput sequence data, which allows a user to easily perform quality control checks on raw sequence data from high-throughput sequencing pipelines. The input data may be BAM, SAM, or FastQ files (any variant). The HTML-based permanent report indicates the problematic areas through summary graphs and tables. FastQC can be installed using Conda.

> conda install fastqc

***Note:*** Bowtie is a super-fast, memory efficient tool for splicing short sequences into template genomes. Bowtie2 can be installed using Conda.

> conda install bowtie2 -c bioconda

***Note:*** Samtools is designed to handle SAM/BAM format alignment files. This tool can sort and merge SAM files and export BAM files. Samtools can be installed using Conda.

> conda install samtools

***Note:*** MACS is one of the most widely used peak-calling programs and can be installed using Conda.

> conda install macs2

3. Install the software required by RNA-seq.

FastQC, STAR, and HTSeq are required for RNA-seq analysis.

***Note:*** The objective of STAR^9^ is to align the sequenced reads to the reference genome. The software is characterised by high accuracy and mapping speed. Notably, the mapping speed is 50 times that of other competitive software.

>conda install STAR

***Note:*** HTSeq is a Python package for analyzing high-throughput sequencing data. HTseq-count is an application that counts the reads in a number of units located on the genome, which refers to a set of locations on the chromosome (commonly known as the gene exon).

>conda install HTSeq

## Key resources table

**Table:** 

REAGENT or RESOURCE	SOURCE	IDENTIFIER
**Software and algorithms**		

Miniconda (v22.9.0)	N/A	https://anaconda.org/anaconda/conda
R (v4.1.0)	N/A	https://www.r-project.org/
Perl (v5.36.0)	N/A	https://www.perl.org/
FastQC (v0.11.9)	Andrews^8^	https://www.bioinformatics.babraham.ac.uk/projects/fastqc
bowtie2 (v2.4.5)	Langmead^1^	https://sourceforge.net/projects/bowtie-bio/
samtools (v1.16.1)	N/A	http://samtools.sourceforge.net/
MACS (v2.1.0)	Zhang Y et al.^3^	https://pypi.python.org/pypi/MACS2
STAR (v2.5.3a)	Dobin et al.^9^	https://github.com/alexdobin/STAR
HTSeq (v2.0.2)	Anders et al.^5^	https://pypi.org/project/HTSeq/
cytoscape (v3.6.1)	Shannon et al.^10^	https://cytoscape.org/
GraphPad Prism (v8.00)	Swift^11^	https://www.graphpad.com/

## Step-by-step method details

### ChIP-seq analysis

**Timing: 30–50 min**

Complete the entire ChIP-seq analysis process, including quality control, alignment and peak-calling. The corresponding test Data can be obtained from Data S1-Methods S1 in the supplemental information.

1. Perform sequence quality control by FastQC.

> conda install fastqc

***Note:*** Major parameters: -o: storage path of the generated report file and file name of the generated report; -t: number of threads; -q progress in real time.

>fastqc ∗.gz

2. Perform alignment.
  ChIP-seq reads are mapped to the *Pseudomonas savastanoi* pv. *phaseolicola* 1448A genome using Bowtie (version 2.4.5). Only the uniquely mapped reads are retained for the subsequent analyses. The test data file can be downloaded from this link: https://github.com/dengxinb2315/STAR-protocol-data.git.
  a. Construct the mapping file.
    ***Note:*** Bowtie is a super-fast, memory efficient tool for splicing short sequences into template genomes. Bowtie2 can be installed using Conda.
    > conda install bowtie2 -c bioconda
    ***Note:*** Mapping the high-throughput data to the index. –threads: number of threads.
    > bowtie2-build --threads 20 Pseudomonas syringae _genome.fa Pseudomonas syringae _genome
    ***Note:*** Build indexes with the FASTA-format P. syringae genome files. –threads: number of threads; -1: the left part of the paired-end data; -2: the left part of the paired-end data; and -S: File for SAM output. More information regarding the genome file can be found here: (NCBI: https://www.ncbi.nlm.nih.gov/data-hub/genome/GCF_000012205.1/ or http://pseudomonas.com/). The corresponding test Data can be obtained from Data S1-Methods S1 in the support file.
    > bowtie2 -p 20 -x *Pseudomonas syringae* _genome -1 R1.fq -2 R2.fq -S Gene_name.bathc1.sam
    ***Note:*** Samtools is designed to handle SAM/BAM format alignment files. This tool can sort and merge SAM files and export BAM files. To install Samtools using Conda, use the command ‘conda install samtools’. The main function of the view command is to convert the input file to the output file. Typically, the SAM file post-alignment is converted to the BAM file, and then various operations are performed on the BAM file. -q: mapping quality >= INT; -bS: output BAM format file and ignore the input format.
    > samtools view -q 255 -bS -> Gene_name.bathc1.bam
  b. Identify the binding peaks (p < 1 × 10^-5^) using MACS (version 2.1.0).
    ***Note:*** MACS is one of the most widely used peak-calling programs. MACS can be installed using Conda with the command ‘conda install macs2’. macs2_callpeak is one of the main macs2 features that can find ChIP-seq peaks using BAM files. -t: treatment group; -c: control group; -f: format of the input file; -g: effective genome size; -n: name of the output file; -B: determines whether the remaining fragment pileup, control lambda, -log10pvalue, and -log10qvalue scores are saved to the bedGraph file; -p: e-value; --nomodel: the bias of the peak candidate region is not considered, and the background λ is used as the local λ; --extsize: length of the transcription-factor-binding region.
    > macs2 callpeak -t Gene_name.batch1.bam -c Input.batch1.bam -f BAM -g 6264404 -n Gene_name_pval -B -p 1e-5 --nomodel --extsize 75
  c. Annotate the peaks (in R)
    ***Note:*** Three R packages are needed. The ChIPpeakAnno package includes functions to retrieve the sequences around the peak; obtain the enriched GO terms; and find the nearest gene, exon, miRNA, or custom features such as the most conserved elements and other transcription-factor-binding sites supplied by users. GenomicFeatures^12^ is a set of tools and methods for preparing and manipulating transcript centric annotations. The GenomicRanges^13^ package defines general purpose containers for storing and manipulating genomic intervals and variables defined along a genome. The test data file can be downloaded from this link: https://github.com/dengxinb2315/STAR-protocol-data.git
    ># In R
    >library(ChIPpeakAnno)
    >library(GenomicFeatures)
    > library(GenomicRanges)
    > ### all peak files###
    > peak_list <- list.files("../3narrowpeak/")
    > ### import annotation files (gene location, obtained from gtf file)
    > sss <- read.delim("∼/workspace/DrDeng/PAVIRnet2019/ReferenceGenome/ ps _NCBI.anno.txt, sep="\t",header=F)
    > sss<-sss[,c(1,4,5,7,9)]
    > colnames(sss)<-c("chr","start","end","strand","id")
    > ge <- as(sss,"GRanges")
    > ### annotation ###
    > for(s in 1 : length(peak_list)){
    pe <- read.delim(paste("../3narrowpeak/",peak_list[s],sep=""),sep="\t",header=F)
    peaks1 <- GRanges(seqnames=as.matrix(pe[,1]),
    ranges=IRanges(start=as.numeric(as.matrix(pe[,2])),
    end= as.numeric(as.matrix(pe[,3])),
    names=as.matrix(as.matrix(pe[,4]))))
    > annotatedPeak <- annotatePeakInBatch(peaks1, AnnotationData=ge)
    > df <- data.frame(as.data.frame(annotatedPeak)
    > as.data.frame(values(ge[as.numeric(values(annotatedPeak)$feature),])))
    > write.table(df,
    paste("1directly_anno/",peak_list[s],"_annotation.txt",sep=""),quote=FALSE, row.names=FALSE, sep="\t")
    > }

### RNA-seq analysis

**Timing: 30–50 min**

Complete the entire RNA-seq analysis process, including quality control, alignment and annotation.

The corresponding test Data can be obtained from Data S2-Methods S2 in the support file.

3. Perform sequence quality control by FastQC.

>fastqc ∗.gz

4. Perform alignment.
  RNA-seq reads are mapped to the *P. syringae* genome using STAR, and only the uniquely mapped reads are retained for the subsequent analyses. The STAR software is designed to align sequenced reads to the reference genome. The software is characterised by high accuracy and mapping speed (50 times those of other competitive software).
  a. Build a genome index.
    ***Note:*** --runMode: type of runs; --runThreadN: number of threads; --genomeDir: index storage location; --genomeFastaFiles: FASTA file for index building.
    > STAR --runMode genomeGenerate --runThreadN 20 –genomeDir STAR_Index_ PS --genomeFastaFiles PS _NCBI_genome.fa
  b. Perform alignment using STAR
    ***Note:*** --runThreadN: number of threads; --genomeDir: index storage location; --readFilesIn: storage location of the paired-end FASTQ files; --outFilterMultimapNmax: maximum number of multiple alignments for a read that will be output to the SAM/BAM files; --outFilterMismatchNmax: alignment is output only if it has no more mismatches than this value.; --outSAMtype: type of SAM/BAM output; --outFileNamePrefix: prefix of the output file; --quantMode: type of quantification requested
    > STAR --runThreadN 60 –genomeDir ./STAR_Index_PS --readFilesIn Gene_name-1_combined_R1.fastq.cutadapt.fastq Gene_name-1_combined_R2.fastq.cutadapt.fastq --outFilterMultimapNmax 1 --outFilterMismatchNmax 2 --outSAMtype BAM Unsorted --
    outFileNamePrefix ./9alignment_batch2/Gene_name_1_ --quantMode GeneCounts

5. Obtain the raw counts using HTSeq.

HTSeq is a Python package for analyzing high-throughput sequencing data. HTSeq-count is an application that counts the reads in a number of units located on the genome, which refers to a set of locations on the chromosome (commonly known as the gene exon).

***Note:*** -f: format of the input file; -r: specifies how the data is sorted; -s: specifies if the data are from a chain-specific library; -t: smallest counting unit; -m: determines whether specific read definitions should be counted when counting reads.

> htseq-count -f bam -r name -s no -t CDS -m intersection-strict Gene_name.bam

PS_gene.gtf > Gene_name_intersection_strict_counts_out.txt

6. Obtain the differentially expressed genes (DEGs).

Obtain the raw counts using DESeq.^14^ The test data file can be downloaded from this link: https://github.com/dengxinb2315/STAR-protocol-data.git

The program outputs the DEG file of the RNA-seq result. In the subsequent analysis, CSV or TXT files can be used.

># In R

> library(DESeq2)

> Gene_name_1 <- read.delim("1counts/Gene_name-1_ReadsPerGene.out.tab",header=F,sep="\t")[- c(1,2,3,4),c(1,2)]

> rownames(Gene_name_1) <- Gene_name_1[,1]

> Gene_name_2 <- read.delim("1counts/Gene_name-2_ReadsPerGene.out.tab",header=F,sep="\t")[- c(1,2,3,4),c(1,2)] >

rownames(Gene_name_2) <- Gene_name_2[,1]

> CTRL_1 <- read.delim("1counts/PS-1_ReadsPerGene.out.tab",header=F,sep="\t")[- c(1,2,3,4),c(1,2)]

> rownames(CTRL_1) <- CTRL_1[,1]

> CTRL_2 <- read.delim("1counts/ PS-2_ReadsPerGene.out.tab",header=F,sep="\t")[- c(1,2,3,4),c(1,2)]

> rownames(CTRL_2) <- CTRL_2[,1]

> mRNA_counts <- cbind(Gene_name_1,Gene_name_2[rownames(Gene_name_1),2], CTRL_1[rownames(Gene_name_1),2],CTRL_2[rownames(Gene_name_1),2])

> mRNA_counts <- mRNA_counts[,-1]

> colnames(mRNA_counts) <- c("Gene_name_1","Gene_name_2","CTRL_1","CTRL_2")

> coldata <-data.frame(name = c("Gene_name_1","Gene_name_2","CTRL_1","CTRL_2"), condition = c("Gene_name","Gene_name","CTRL","CTRL"))

> cds <- DESeqDataSetFromMatrix(countData = dds, colData = coldata, design = ∼condition)

> mat <- mRNA_counts[,c("CTRL_1","CTRL_2","Gene_name_1", "Gene_name_2")]

> dds <- mat[ rowSums(mat==0) < 3, ]

> coldata <-data.frame(name = c("CTRL_1","CTRL_2","Gene_name_1","Gene_name_2"), condition = c("ctrl","ctrl","Gene_name","Gene_name"))

> cds <- DESeqDataSetFromMatrix(countData = dds, colData = coldata, design = ∼condition)

> colData(cds)$condition <- relevel(colData(cds)$condition , "ctrl")

> dds <- DESeq(cds)

> Gene_name_res <- results(dds)

> Gene_name_DEGs_adjp <- Gene_name_res[intersect(which(abs(Gene_name_res$log2FoldChange) >1),which(Gene_name_res$padj < 0.05)),]

> write.csv(Gene_name_DEGs_adjp,file="Gene_name_DEGs.csv")

>

### Statistical results and visualization of the RNA-seq data

**Timing: 30–50 min**

Complete statistical analysis and visualization of RNA-seq data.

7. Prepare the stacked bar of DEGs.
  The stacked bar is prepared using GraphPad Prism^11^ (version 8.00), based on the DEG data. First, the numbers of downregulated and upregulated DEGs in each mutant are counted. A typical process involves the following steps.
  a. Create a new project file by double clicking the gray area of the window or the little triangle of Prism. Select the table type Grouped in the pop-up wizard window.
  b. Copy and paste the data into the Prism table, transpose the data matrix, click the Analyse button, and select Transpose X and Y as the calculation method.
  c. Click the newly generated chart under Graphs and select the corresponding chart type. The stacked chart is Grouped under Summary Data.
8. Make personalized adjustments to the chart, such as the colors or thickness of the axes. Establish the co-expression network.
  a. Establish the regulatory network using Cytoscape^10^ (version 3.6.1), based on the numbers of downregulated and upregulated DEGs in each strains.
    i. Build a data matrix, in which the first column lists the names of the strains, and the second column lists the corresponding DEGs.
    ii. Open Cytoscape and import the network by file. Select the first and second columns as the source and target nodes, respectively.
  b. Make personalized adjustments to the chart, for instance, enclose the upregulated targets and downregulated genes in red and blue circles, respectively.

***Note:*** The size of mutant circles indicates the number of DEGs. The layout is set as the Grid Layout.

### Overview of ChIP-seq data

**Timing: 30–50 min**

Complete statistical analysis and visualization of ChIP-seq data.

9. Determine the number of target genes per cluster.
  a. The ChIP-seq data are classified into five clusters (NtrC-like, LytR-like, OmpR-like, NarL-like, and PrrA-like). The corresponding test Data can be obtained from Data S3-Methods S3 in the support file.
  b. The number of target genes in the clusters is counted and visualized using GraphPad Prism (version 8.00). See the description above for detailed steps.
10. Derive the Venn plot of the gene targets between clusters.
  a. The overlapping target genes are shown in a Venn plot derived using the VennDiagram^15^ package in R (version 4.1.0).
  b. The target genes of each cluster are incorporated in one file, in which each column contains the name of the target gene.
  c. The test data file can be downloaded from this link: https://github.com/dengxinb2315/STAR-protocol-data.git. The R code for the Venn plot is as follows:

>##install.packages("VennDiagram") ###run this code when the first time.

> library(VennDiagram)

> Venn_data <- read.table(“venn_data.txt”, header=T,row.names=1, sep = “\t”)

> Venn_data <- data.frame(Venn_data) > venn.diagram(x = list(NtrC-like, LytR-like, OmpR-like, NarL-like, PrrA-like),

category.names = c("NtrC-like ", " LytR-like ", " OmpR-like "," NarL-like "," PrrA-like"),

filename = 'Venn_diagramm.png',

output=TRUE,

#Output picture parameters, including picture type, height and width, resolution, compression and so on.

imagetype="png",

height = 1000,

width = 1000,

resolution = 300,

compression = "lzw",

#The setting of the ring includes the width of the edge, whether a dotted line is required, and the color of the fill.

lwd = 2,

lty = 'blank',

fill = c("#EE3B3B", "#6495ED", "#8B7355", "#EEC900", "#008B8B"),

#Setting the number on the graph includes size, boldness, and font

cex = .6,

fontface = "bold",

fontfamily = "sans",

#Adjust the name of each collection, including font size, whether bold, on the outside, as well as location, font and its color

cat.cex = 0.7,

cat.fontface = "bold",

cat.default.pos = "outer",

cat.pos = c(0,-45,240,135,45),

cat.dist = c(0.2,0.2,0.2,0.2,0.2),

cat.fontfamily = "sans",

cat.col=c("#EE3B3B", "#6495ED", "#8B7355", "#EEC900", "#008B8B"))

11. Obtain the histogram and heatmap to clarify the number of genes uniquely targeted by mutants
  a. In the case of more than five datasets, it is difficult to promptly extract useful information directly from the Venn plot.
  b. Use the R package UpSetR^16^ to explore the number of genes uniquely targeted in multiple groups. First, integrate the peaks of each cluster into one file, in which each column contains all of the peaks of one sample.
  c. The R code for the Venn plot is as follows:

>##install.packages(UpSetR) ###run this code when the first time.

> library(UpSetR)

> data<-read.table('upsetR_data.txt',header=T,sep="\t",row.names=1)

> Upset(data,

nset = 8,

nintersects = 30,

order.by = c('freq', 'degree'),

decreasing = c(TRUE, TRUE),

queries = list(list(query = intersects, params = 'c1', color = '#00A087B2'),

list(query = intersects,

params = c('3220', 'GacA', '1374', '0642', '1364', '1906', ‘4419’, ‘3800’),

color = 'darksalmon')))

### Regulatory network and functional heatmap

**Timing: 1–2 days**

The calculation of relevant coincidence sites and establishing network for integrating ChIP-seq and RNA-seq data.

12. Construct the regulatory network.
  a. The log2 fold change (log2FC) of the DEGs of each mutant is extracted based on the RNA-seq data.
  b. Cytoscape is used to construct the regulatory network.
  c. The format of the input file is the classic network style, which includes the mutant names in the first column, DEG names in the second column, and log2FC in the third column.
  d. Use Cytoscape to build the networks through the following steps:
    i. Open Cytoscape and import the network by file.
    ii. Select the first and second columns as the source and target nodes, respectively.
    iii. Make personalized adjustments to the chart, for instance, enclose the upregulated targets and downregulated genes in red and blue circles, respectively.
    iv. The size of the mutant circles represents the number of regulons.
    v. The layout is set as the Circular Layout.
13. Construct the functional heatmap.

Select the DEGs regulated by the cluster mutants to perform the GO analysis. First, use the in-house Perl script to determine the GO terms.

>#!/usr/bin/perl

> @a = glob("./∗.txt");

> open OUT,">../ID.txt";

> foreach $a (@a)

>{

> open IN,"$a"

> readline(IN);

> while($line=<IN>)

> {

>   chomp $line;

>  @line =split(/\t/,$line);

>   if($hash{$line[0]})

>  {

>    next;

>  }

>   else.

>  {

>   $hash{$line[0]} = 1;

>  }

>  }

>}

> foreach $key (keys %hash)

>{

>  print OUT "$key\n";

>}

Second, combine all of the p-values of each mutant into one matrix using the in-house Perl script.

>#!/usr/bin/perl

> open IN,"../ID.txt";

> open OUT,">../TEST.txt";

> while($line=<IN>)

>{

>  chomp $line;

>  $hash{$line} = 1;

>}

>@a = glob("∗.txt");

> foreach $a (@a)

>{

>   $a =∼ /(\S+).txt/;

>  $name = $1;

>   open IN,"$a";

>  readline(IN);

>   while($line=<IN>)

>  {

>    chomp $line;

>    @line = split(/\t/,$line);

>    $hash{$line[0]}.= "∗$name-$line[1]";

>  }

>   foreach $key (keys %hash)

>  {

>    if($hash{$key} =∼ /\∗$name/)

>   {

>     next;

>   }

>    else

>   {

>    $hash{$key}.= "∗$name-NA";

>   }

>  }

> foreach $key (keys %hash)

>{

>   print OUT "$key\t$hash{$key}\n";

>}

To obtain the final data for constructing the heatmap, transfer the p-values of the GO terms to -log_10_(p-value) through an in-house script.

>#!/usr/bin/perl

> open IN,"C:\\Users\\22896\\Desktop\\DYQ_go.txt";

> open OUT,">C:\\Users\\22896\\Desktop\\DYQ_go_log.txt";

> #$a = readline(IN);

> #print OUT "$a";

> while($line=<IN>)

>{

>    chomp $line;

>   @line = split(/\t/,$line);

>   foreach $lin (@line)

>   {

>     if($lin == 0)

>    {

>      print OUT "$lin\t";

>    }

>     else

>    {

>     $a = -(log($lin)/log(10));

>     print OUT "$a\t";

>    }

>  }

>  print OUT "\n";

> }

Finally, construct the functional heatmap using the R package pheatmap.^17^ The R code is as follows:

> #install.packages(“pheatmap”)

> setwd("C:\\Users\\22896\\Desktop")#set the working path

> library(pheatmap) > data<-read.table("yhy.txt", row.names=1, header=T, sep = "\t")

> pheatmap(data,scale="row",border_color="black",color=colorRampPalette(rev(c("red","black","green")))(102),width=10,height=5,show_rownames=F)

## Expected outcomes

First, the DEGs are identified using DESeq2 based on the RNA-seq data. Second, the binding peaks are identified after mapping the ChIP-seq reads to the genome files. Additional information, such as the sequences around the peak; enriched GO terms; and nearest gene, exon, miRNA, or custom features, is obtained through annotation. Third, the network of all interactions involving mutant target genes is construct based on the ChIP-seq results. Finally, the mutant regulatory network is established by integrating the regulons of the cluster mutants from the ChIP-seq and RNA-seq data obtained in different medium conditions.

## Limitations

This version of the protocol is based on the RNA-seq and ChIP-seq data of bacteria that possess small genomes. Although it may be effective for other species, it may require considerable computing resources and long runtimes when processing data with huge genome files.

## Troubleshooting

### Problem 1

Software installation through Conda is extremely slow (Related to the section ‘Install the required software’).

### Potential solution

• Update the Conda software to the latest version using the command ‘conda update conda’.
• Install the software using MAMBA instead of Conda. First, use the command ‘conda install mamba’ and then ‘mamba install software’.

### Problem 2

The substrate language conflicts when Conda is used to directly install software. (Related to the section ‘Install the software required by ChIP-seq’)

### Potential solution

Different programs may incorporate different versions of Python. To solve this problem, a special environment should be created for the software with the command ‘conda create -n env-name soft-name=version’.

### Problem 3

Cannot install the R package in R. (Related to the section ‘Install R’)

### Potential solution

• Install the R version according to the requirements of the package.
• Use BiocManager to install the package: install.packages("BiocManager") && BiocManager::install("Package-name").
• Use devtools to install the package: install.packages("devtools") && install_github ("author-name /Package-name").
• Using devtools to install the package: install.packages("devtools") && install_github ("author-name /Package-name").

### Problem 4

Errors appear when using ‘read.table’ in R. (Related to the section ‘Obtain the histogram and heatmap to clarify the number of genes uniquely targeted by mutants’)

### Potential solution

• Check whether each column in the data is tab-separated.
• Check the line name for special symbols or spaces.
• Check data integrity (presence of empty rows or redundant data).

### Problem 5

The program yields too many DEGs based on the RNA-seq data, rendering the results chaotic and disordered. (Related to the section ‘RNA-seq analysis’)

### Potential solution

Adjust the screening criteria to obtain the appropriate number of DEGs. When too many DEGs are identified, log2FC can be increased to 2 or the p-value can be decreased to 0.01.

## Resource availability

### Lead contact

Further information and requests for resources and reagents should be directed to and will be fulfilled by the lead contact, Xin Deng (xindeng@cityu.edu.hk).

### Materials availability

This study did not generate new unique reagents.

### Data and code availability

This study did not generate new datasets or codes.

## Supplemental information

Data S1. Intermediate file for ChIP-seq analysis, related to Step: Perform alignment

Data S2. Intermediate file for RNA-seq analysis, related to Step: Perform alignment using STAR

Data S3. Intermediate file for overview of ChIP-seq data, related to Step: Determine the number of target genes per cluster
